# H^+^-ATPase blockade reduced renal gluconeogenesis and plasma glucose in a diabetic rat model

**DOI:** 10.1007/s00795-017-0175-6

**Published:** 2018-01-09

**Authors:** Akihiro Tojo, Saaya Hatakeyama, Masaomi Nangaku, Toshihiko Ishimitsu

**Affiliations:** 1 Department of Cardiology and Nephrology, Dokkyo Medical University, 880 Kitakobayashi, Mibu, Tochigi 321-0293 Japan; 2 Division of Nephrology and Endocrinology, The University of Tokyo, Tokyo, Japan

**Keywords:** Ammoniagenesis, Diabetes mellitus, Gluconeogenesis, Vacuolar-type proton ATPase, Sodium glucose co-transporter

## Abstract

Vacuolar H^+^-adenosine triphosphatase (ATPase) plays important roles in urinary acid excretion, vesicular acidification to activate enzymes, and the membrane recycling of transporters in the kidney. As acidosis stimulates renal gluconeogenesis, we investigated the effect of blockade of H^+^-ATPase on renal gluconeogenesis in diabetic rats. Diabetes mellitus was induced by a single injection of streptozotocin, and a group of DM rats was treated with bafilomycin B1 intraperitoneally for 8 days. In diabetic rats, the renal expression and activity of H^+^-ATPase were increased with elevated urinary ammonium excretion. The blockade of H^+^-ATPase by bafilomycin B1 reduced the renal H^+^-ATPase activity and urinary ammonium excretion in diabetic rats. Treatment with bafilomycin suppressed the enhancement of the renal gluconeogenesis enzymes phosphoenol pyruvate carboxykinase and glucose-6-phosphatase in diabetic rats and reduced the renal cytoplasmic glucose levels, whereas hepatic gluconeogenesis did not change significantly. After a 24-h starvation period, bafilomycin decreased the plasma glucose level to a normal level in diabetic rats. The suppression of renal gluconeogenesis by an H^+^-ATPase inhibitor may therefore be a new therapeutic target for the treatment of diabetes mellitus.

## Introduction

Renal gluconeogenesis is mainly regulated by acidosis and starvation. Its proportion increases from 10% of total gluconeogenesis under feeding conditions to 40–50% after starvation [1–3]. The main substrates for renal gluconeogenesis are lactate (from the muscle) and glutamine (from throughout the body) [3–5]. Glutamine is converted to glutamate and then to α-ketoglutarate in the mitochondria by a deaminase reaction, producing two ammonia molecules in a process called ammoniagenesis. Then, α-ketoglutarate enters the tricarboxylic acid (TCA) cycle as a source of gluconeogenesis [6]. Phosphoenol pyruvatecarboxykinase (PEPCK) is activated by acidosis in the kidneys of rats fed ammonium chloride, indicating that acidosis enhances renal gluconeogenesis [6–8].

Vacuolar H^+^-adenosine triphosphatase (ATPase) is expressed in the brush border membrane of the proximal tubules and in the intercalated cells of the collecting duct to play an important role in the acid–base balance [9–12] as well as in endocytosis by the acidification of endocytic vesicles [12–14]. The proximal convoluted tubules are the most important sites for both renal ammoniagenesis and gluconeogenesis, and acidosis promotes the urinary excretion of ammonium through the activation of H^+^-ATPase and gluconeogenesis [6–8]. A specific inhibitor of H^+^-ATPase, bafilomycin (BFM) B1, was discovered from Streptomyces by Nobel Laureate Professor Satoshi Omura as setamycin in 1981 [15]. We hypothesized that inhibition of H^+^-ATPase by BFM B1 may inhibit renal gluconeogenesis and could reduce fasting plasma glucose level under starvation condition.

## Materials and methods

### Animal experiments

Sprague–Dawley rats weighing 180–200 g (Charles River Laboratories, Shizuoka, Japan) had ad libitum access to tap water and standard rat chow. Diabetes was induced by a single tail vein injection of streptozotocin (STZ, 60 mg/kg body weight; Sigma Chemical, St. Louis, MO, USA) [diabetes mellitus (DM) rats]; the control rats were injected with an equal volume of citrate buffer. Three weeks after STZ injection, a group of DM rats was treated with BFM B1 (50, 100, 200 nmol/kg/day intraperitoneally; Enzo Life Sciences, Ann Arbor, MI, USA). Twenty-four-hour urine and blood samples were collected using a metabolic cage until day 7 morning under feeding condition with free access to water and food, and then under 24-h starvation conditions without food [16]. On day 8, the rats were anesthetized with pentobarbital (50 mg/kg body weight), and then their kidneys and liver were removed and used for western blotting or immunohistochemistry. Intravenous insulin tolerance tests (ITTs) were performed to assess the degree of insulin resistance, and the extent of insulin resistance was evaluated according to the K index of ITT (KITT) as described previously [16].

All the procedures were conducted in accordance with the Guidelines for Animal Experiment and Ethics Committee in The University of Tokyo (P10-079, 15-P-134), and Dokkyo Medical University (17-918).

### Western blotting

As described previously [16], kidneys were homogenized in a fivefold volume of 20 mmol/L Tris buffer with proteinase inhibitors. After centrifugation at 5,000*g* for 15 min, the supernatants were centrifuged at 48,000g for 60 min at 4 °C to obtain the cytosolic fraction and membrane fraction. The 50 µg of renal proteins were applied to a 4–20% gradient gel and electroblotted onto polyvinylidene fluoride membranes. The specific protein bands were identified using rabbit polyclonal antibodies for sodium glucose co-transporter 2 (SGLT2) (Abcam, Tokyo, Japan), G6Pase (Abcam), or PEPCK (Abcam) at 1:500 dilution followed by a horseradish peroxidase-conjugated secondary antibody against rabbit immunoglobulin G (Dako, Glostrup, Denmark) and the bands were visualized with diaminobenzidine reaction. The band stained with anti-beta-actin antibody (Abcam) was used as a loading control. The density of the bands was analyzed using the National Institutes of Health Image software program (version 1.63).

### Immunohistochemistry

Immunohistochemistry was performed using wax sections (2-µm-thick) with polyclonal antibody against SGLT2 (Abcam) or PEPCK (Abcam) or monoclonal antibody against vacuolar-type H^+^-ATPase B2 isoform (Santa Cruz Biotechnology, CA, USA) at 1:200 dilution, and immunoreactivity was detected by horseradish peroxidase reaction as previously described [16].

### Measurement of glucose, hemoglobin A1c, glucose-6-phosphate, glucagon, ammonium, and H^+^-ATPase activity

The glucose levels in the blood, urine and the renal and hepatic cytosolic fraction were measured using the Glutest Pro R device (Arkray Factory, Shiga, Japan). The hemoglobin A1c (HbA1c) level was measured using a DCA 2000 Plus system (Bayer Medical, Tokyo, Japan). The glucose-6-phosphate levels in the homogenates of renal and hepatic cytosolic fraction were measured with the Glucose-6-Phospate assay kit (Abcam). Plasma glucagon was measured by RIA 2 antibody methods. Ammonium was measured using a PocketChem BA PA-4140 (Arkray Factory). The H^+^-ATPase activity in the kidney homogenate was measured using the *N*-ethylmaleimide-sensitive ATPase assay, as previously described [17].

### Statistical analysis

The data are expressed as mean ± standard deviation. A Kruskal–Wallis analysis with a Steel–Dwass post hoc test was used for the comparison among groups. Values of *p* < 0.05 were considered to indicate statistical significance.

## Results

### Renal expression of H^+^-ATPase and ammonium excretion in diabetic rats

**Fig. 1:**
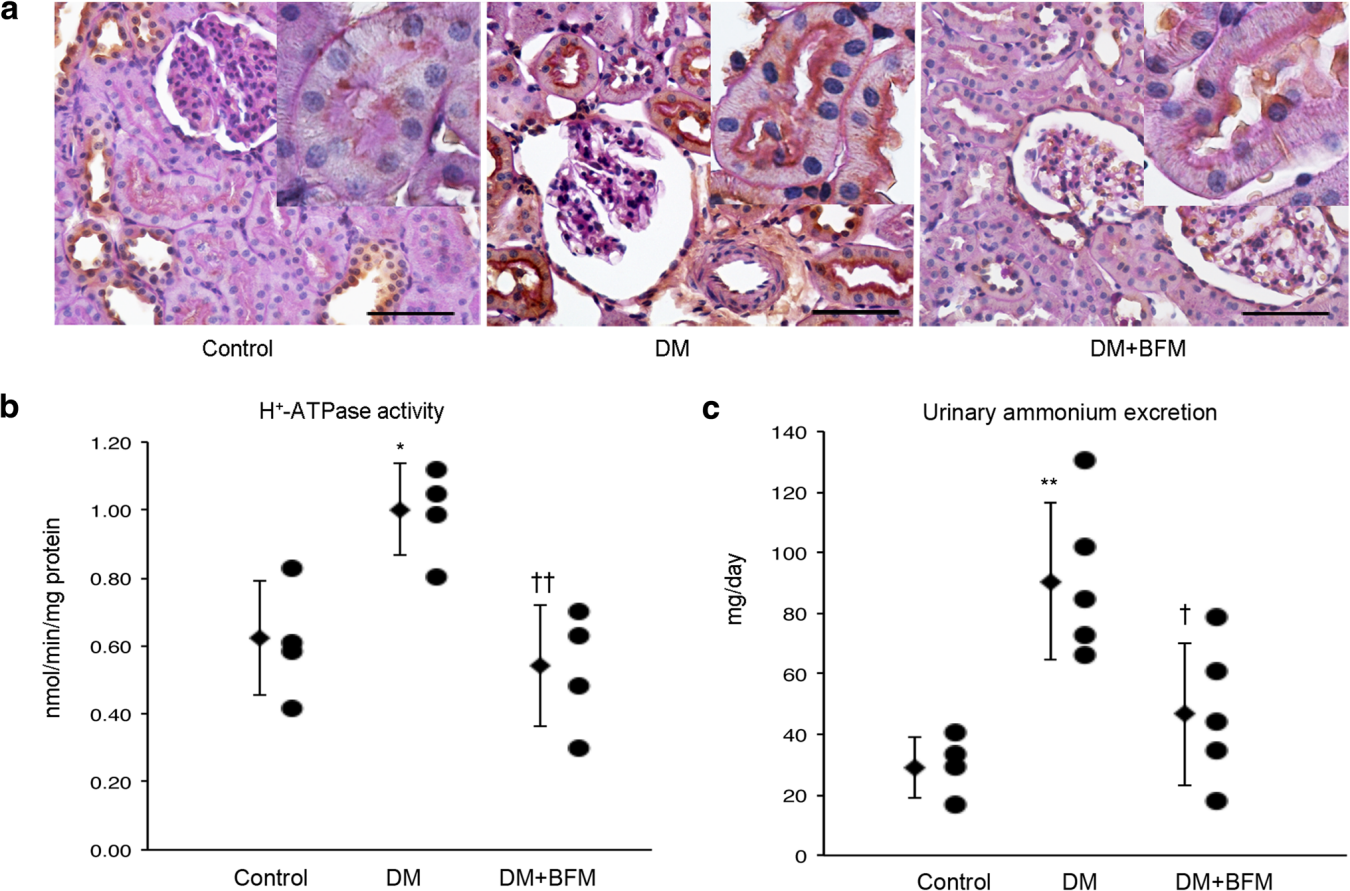
The H^+^-ATPase expression in the proximal tubule (**a**) and its activity in the kidney (**b**), and the urinary ammonium excretion (**c**). Diabetes mellitus (DM), streptozotocin-induced diabetic rats; and DM + bafilomycin (BFM), DM rats treated with bafilomycin B1 at a dose 100 nmol/kg body weight. Inset: a high magnification of proximal tubule expressed H^+^-ATPase in the brush border membrane. *n* = 4–5, *p < 0.05, **p < 0.01 vs. control, ^†^*p* < 0.05, ^††^*p* < 0.01 vs. DM. The bar indicates 100 µm

We identified H^+^-ATPase in the brush border membrane of the proximal tubule and its expression (Fig. 1a) and activity in the kidney (Fig. 1b) were increased in DM rats in comparison with that of control rats. Because of H^+^-ATPase activation, urinary ammonium excretion in the urine of diabetic rats was increased in comparison with that of control rats (Fig. 1c). The diabetic rats treated with BFM showed suppressed renal H^+^-ATPase activity and urinary ammonium excretion level.

### Renal gluconeogenesis and SGLT2 increase the cytoplasmic glucose level

**Fig. 2:**
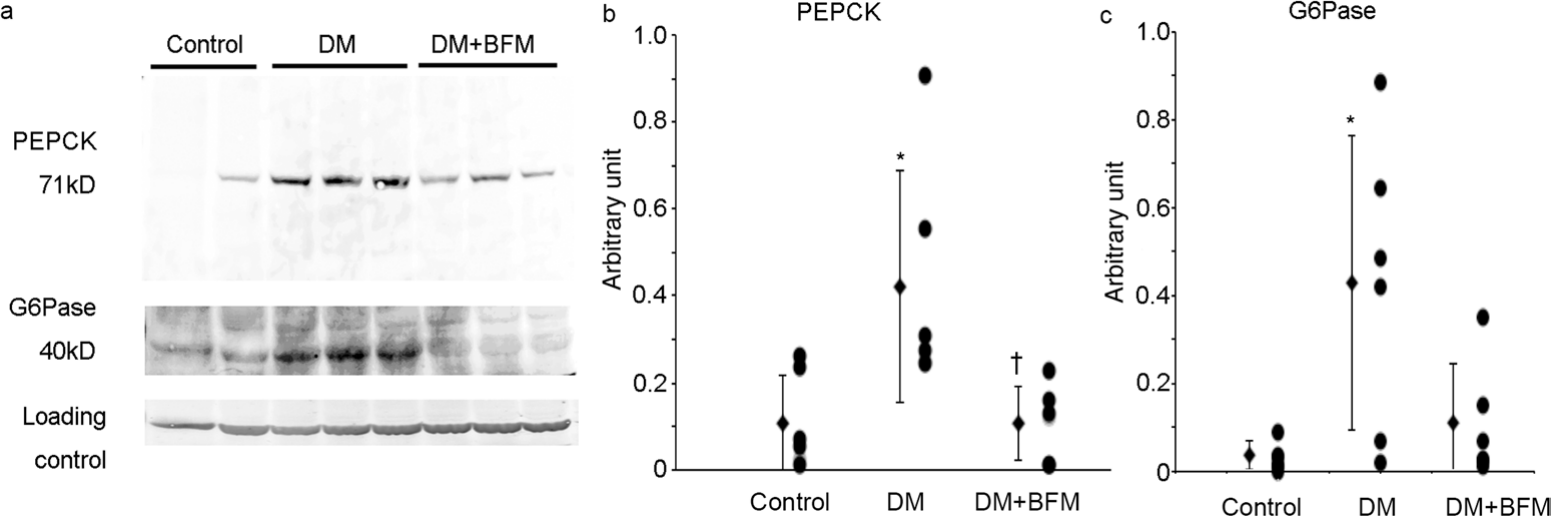
Renal gluconeogenesis enzymes. **a** Western blotting and their densitometry of **b** phosphoenol pyruvate carboxykinase (PEPCK) and **c** glucose-6-phosphatase (G-6-Pase) in the kidney homogenate. Each *n* = 6, *p < 0.05 vs. control, ^†^*p* < 0.05 vs. diabetes mellitus (DM)

**Fig. 3:**
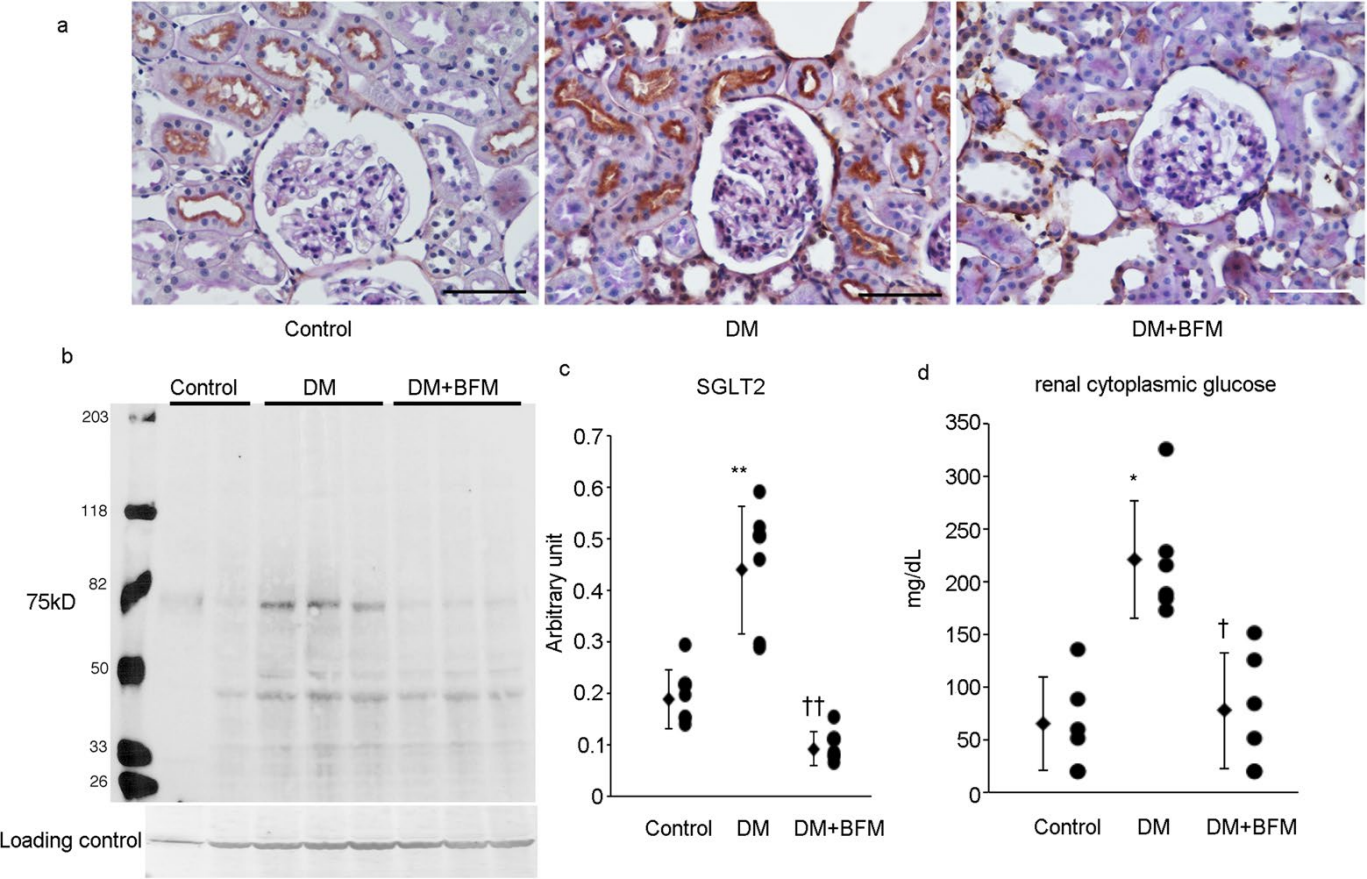
Renal sodium glucose co-transporter 2 (SGLT2) and renal cytoplasmic glucose. Immunostaining (**a**) and western blot analysis (**b, c**) for SGLT2, and renal cytoplasmic glucose level (**d**). Diabetes mellitus (DM), streptozotocin-induced diabetic rats; and DM + bafilomycin (BFM), DM rats treated with bafilomycin B1 at a dose 100 nmol/kg body weight. Each *n* = 6, *p < 0.05, **p < 0.01 vs. control, ^†^*p* < 0.05, ^††^*p* < 0.01 vs. DM. The bar indicates 100 µm

Renal expression of the gluconeogenesis enzymes PEPCK, and glucose-6-phosphatase was increased in the kidney (Fig. 2), which is consistent with our previous data [16]. The blockade of H^+^-ATPase by BFM suppressed the enhanced PEPCK in the diabetic kidney, and glucose-6-phosphatase expression in the kidney treated with BFM did not differ from that in the control kidney (Fig. 2). The renal expression of SGLT2 was increased in the brush border membrane of the proximal tubule of diabetic rats in comparison with control rats (Fig. 3). This led to the increased reabsorption of glomerular filtrated glucose in the proximal tubule. The SGLT2 expression in the proximal tubule was significantly reduced by BFM treatment (Fig. 3).

Because of the increased levels of gluconeogenesis enzymes and SGLT2 expression in the kidney of diabetic rats, the renal cytoplasmic glucose levels were significantly increased in the diabetic rats, which was significantly reduced by BFM treatment (Fig. 3d).

### Hepatic gluconeogenesis

**Fig. 4:**
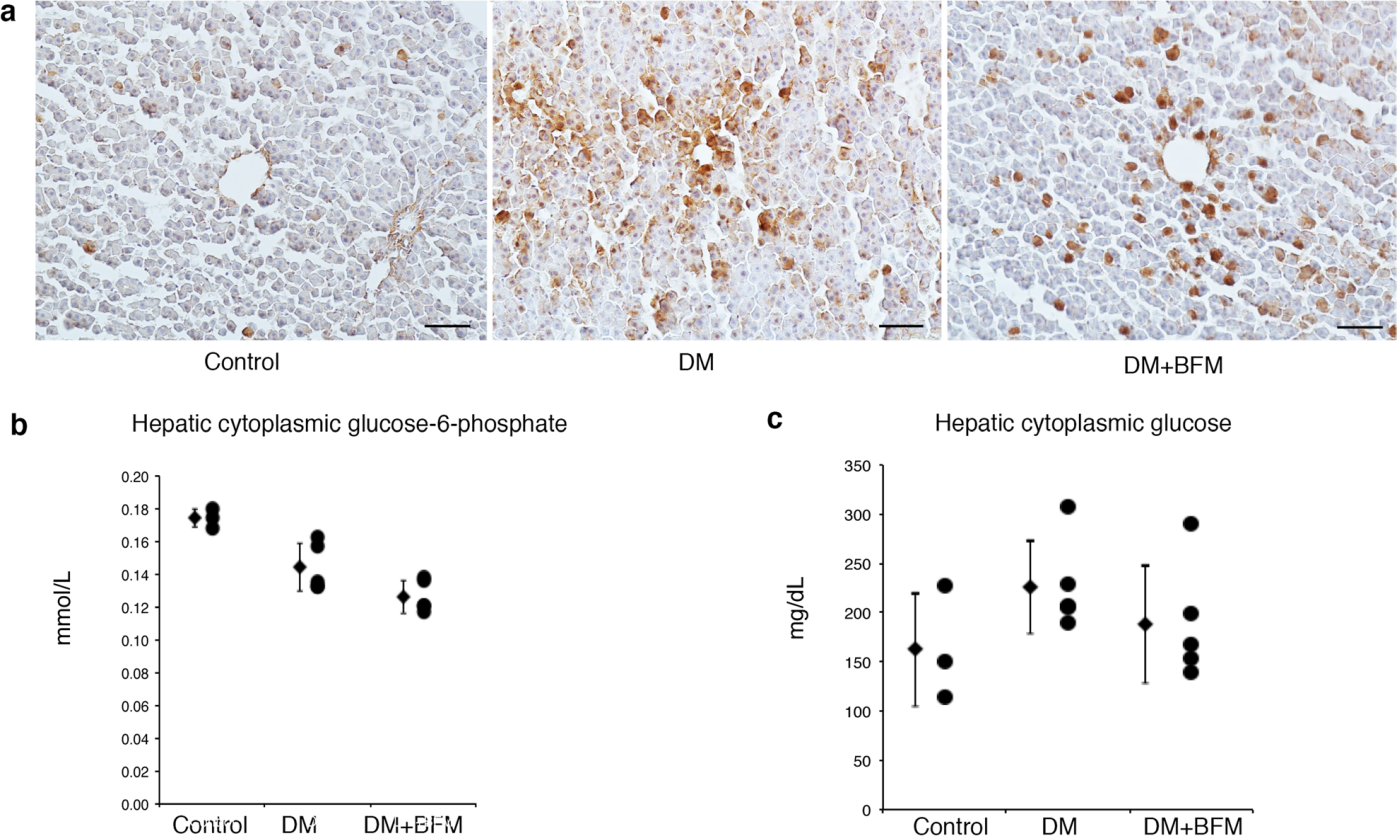
The effects of bafilomycin B1 on hepatic gluconeogenesis. **a** Immunohistochemistry for phosphoenol pyruvate carboxykinase (PEPCK) in the liver. The hepatic glucose-6-phosphate (**b**) and glucose (**c**) levels in the cytosolic fraction of liver homogenates. Diabetes mellitus (DM), streptozotocin-induced diabetic rats (*n* = 5); DM + bafilomycin (BFM), DM rats treated with bafilomycin B1 at a dose 100 nmol/kg body weight (*n* = 5); and control (*n* = 3). The bar indicates 50 µm

The immunoreactivity of hepatic PEPCK, was increased in the liver of STZ-induced diabetic rats. Given that hepatic PEPCK levels are increased by cortisol and glucagon and decreased by insulin but not regulated by acidosis, the blockade of H^+^-ATPase by bafilomycin did not induce a significant reduction in the plasma glucagon level (Table 1), or in the hepatic PEPCK expression (Fig. 4a), hepatic cytoplasmic glucose-6-phosphate (Fig. 4b) or glucose levels (Fig. 4c) after 24-h starvation condition.

**Table 1:** The changes in body weight, food intake, plasma and urinary glucose, and HbA1c under feeding conditions (D0 and D7) or after 24 h fasting (D8)

	Control (*n* = 6)	DM (*n* = 6)	DM + BFM 100 nmol/kg (*n* = 6)
Body weight (g)			
D0	359 ± 57	272 ± 32	265 ± 37
D7	361 ± 39	277 ± 25	275 ± 32
Food intake (g)			
D0	10 ± 4	30 ± 6	27 ± 5
D7	18 ± 2	42 ± 2**	19 ± 3^††^
Urinary volume (mL/h)			
D0	0.6 ± 0.1	3.5 ± 1.0	4.8 ± 1.5*
D7	0.5 ± 0.1	5.0 ± 1.0*	1.7 ± 0.5^†^
Urinary glucose excretion (mg/day)			
D0	8 ± 1	18,954 ± 3466**	17,706 ± 2093**
D7	5 ± 3	21,275 ± 1125*	12,358 ± 5255
Plasma glucose (mg/dL)			
D0	94 ± 11	503 ± 38**	508 ± 37**
D7	100 ± 8	509 ± 32**	329 ± 128**
HbA1c %			
D0	3.6 ± 0.1	7.3 ± 0.4**	7.5 ± 0.5**
D7	4.0 ± 0.2	8.9 ± 0.6**	7.9 ± 0.3**
Plasma glucagon (pg/mL)			
D8	280 ± 44	256 ± 93	243 ± 72
KITT value (each *n* = 3)			
D8	7.8 ± 1.5	1.0 ± 0.7**	4.4 ± 0.3*^,†^

*BFM* bafilomycin; *D0* day 0, *D7* day 7, *D8* day 8, *DM* diabetes mellitus, *HbA1c* hemoglobin A1c

*p < 0.05, **p < 0.01 vs. control, ^†^*p* < 0.05, ^††^*p* < 0.01 vs. DM

### Plasma glucose levels and urinary glucose excretion after 24-h starvation

**Fig. 5:**
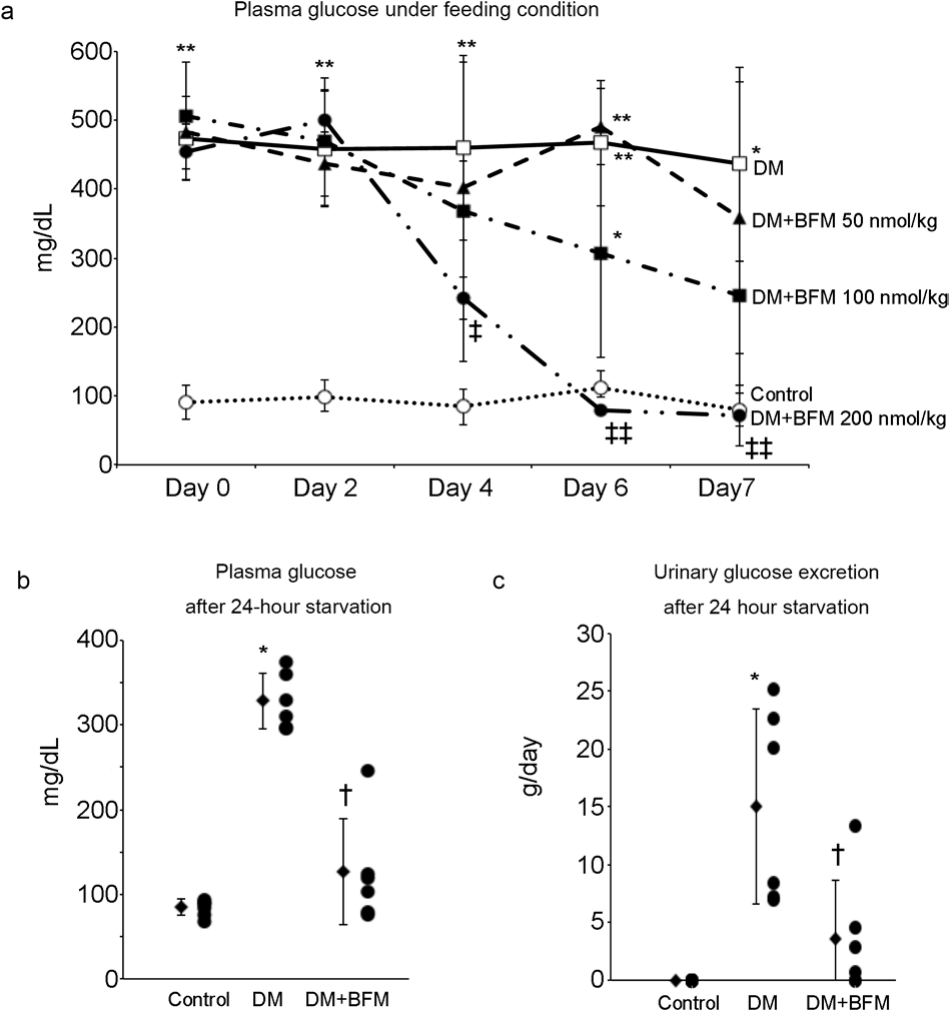
The effects of bafilomycin (BFM) B1 on the plasma glucose and urinary glucose excretion. **a** The time course and dose-dependent effects of BFM on plasma glucose under feeding condition. Control rats (*n* = 7); DM, streptozotocin-induced diabetic rats (*n* = 8); DM + BFM 50 nmol/kg, DM rats treated with bafilomycin 50 nmol/kg body weight (*n* = 3); DM + BFM 100 nmol/kg, DM rats treated with bafilomycin 100 nmol/kg body weight (*n* = 5); DM + BFM 200 nmol/kg, DM rats treated with bafilomycin 200 nmol/kg body weight (*n* = 3). Plasma glucose level (**b**) and urinary glucose excretion (**c**) after 24-h starvation. Control (*n* = 6), DM (*n* = 6), DM + BFM at a dose 100 nmol/kg body weight (*n* = 6). *p < 0.05, **p < 0.01 vs. control, ^†^*p* < 0.05 vs. DM, ^‡^*p* < 0.05, ^‡‡^*p* < 0.01 vs. day 0

The blockade of H^+^-ATPase by BFM B1 decreased plasma glucose levels in a dose-dependent manner in STZ-induced diabetic rats (Fig. 5a). Prior to the start of treatment with BFM B1, body weight, amount of food intake, urinary volume (reflecting water intake), plasma glucose and HbA1c of the DM and DM + BFM groups were the same (Table 1). After 7 days of treatment with BFM B1, the amount of food intake and urinary volume were decreased; however, there was no significant change in body weight (Table 1). When DM rats were under feeding conditions, BFM B1 (100 nmol/kg body weight) reduced their plasma and urinary glucose levels by about 30–40% of the pretreatment values, however the differences did not reach statistically significant (Table 1). Interestingly, the hypoglycemic effect of BFM became more obvious and statistically significant after 24-h starvation conditions (Fig. 5b, c). This is an insulinopenic model of diabetes with degraded pancreatic islet cells by radicals produced by STZ, and plasma glucagon level did not change significantly with BFM treatment. The insulin sensitivity evaluated by KITT was decreased in DM and it recovered significantly with BFM treatment (Table 1).

## Discussion

In this study, we demonstrated that the H^+^-ATPase activity and ammoniagenesis are enhanced in diabetic rats. Thus far, the H^+^-ATPase activity in the kidney has not been studied in DM models. Contrary to our results, the H^+^-ATPase activity in the microvascular endothelial cells was found to be decreased in a diabetic model [18]. Given that prorenin receptors stimulate the H^+^-ATPase activity in the renal tubular cells [19] and that the level of prorenin receptors is increased in the diabetic kidney [20], our finding of enhanced H^+^-ATPase activity in the kidneys of diabetic rats seems reasonable.

The hypoglycemic effect of the blockade of H^+^-ATPase by BFM B1 is quite striking, as it is effective in an animal model of STZ-induced insulinopenia. Thus, the anti-diabetic effect was not ascribed to insulin secretion or insulin sensitivity, even though the reduced insulin sensitivity evaluated by KITT value in STZ-diabetic rat was increased by BFM B1 treatment. Also plasma glucagon level and hepatic gluconeogenesis did not change significantly by BFM B1 treatment, so the main mechanism of the hypoglycemic effect of BFM may be dependent upon renal gluconeogenesis under starvation condition. More than 30 years ago, it was reported that vanadate reduced plasma glucose levels in diabetic rats through the inhibition of increased hepatic and renal levels of PEPCK, tyrosine aminotransferase, and glucokinase [21–24]. Interestingly, vanadate is an inhibitor of P-type ATPase, but also inhibits H^+^-ATPase [25–27] and this could be related to the hypoglycemic effect of vanadate. Furthermore, chloroquine inhibits H^+^-ATPase and glucose formation in the liver and the kidney through the suppression of PEPCK and G-6-Pase [28]. The hypoglycemic effect of chloroquine is blocked by NH_4_Cl [28], which induces metabolic acidosis and stimulates ammoniagenesis and H^+^-ATPase. Metabolic acidosis stimulates PEPCK and gluconeogenesis in the kidney [29, 30]. These reports sport our hypothesis that the inhibition of H^+^-ATPase reduces renal gluconeogenesis and plasma glucose level under starvation in diabetic rats. Acidosis and starvation enhances renal gluconeogenesis enzymes but not hepatic gluconeogenesis. The later is mainly regulated by insulin, glucagon and cortisol [1–3, 29, 30]. Thus, H^+^-ATPase blockade by BFM significantly reduced renal PEPCK, but not hepatic PEPCK expression.

In the present study, BFM showed antidiabetic effects in insulin-depleted STZ diabetic rats without significant changes in the body weight or blood pressure. As vacuolar H^+^-ATPase is also expressed in the various cells including osteoclast, lung, testis and neuroendocrine cells [12, 14], thus, it is possible that antidiabetic effects of BFM could be dependent upon the blocking effect on the non-renal cells. Recently it has been reported that adult mice with the conditional ablation of *Atp6ap* demonstrated a significant reduction of plasma glucose, however, they also showed abnormalities in the intestine and hematopoietic cells [31]. The limitation of this study is that one STZ rat treated with 200 nmol/kg body weight of BFM died during 24-h fasting because of hypoglycemia. Prof. Omura reported that all the mice survived with 0.6 mg/kg of setamycin, whereas more than 1.25 mg/kg of setamycin killed them [15]. The reduction of food intake could be related to toxicity of BFM, and further studies are necessary to clarify the safety of BFM with longer periods of treatment.

### Conclusion

The suppression of H^+^-ATPase by bafilomycin reduced renal gluconeogenesis and SGLT2 expression in the proximal tubules and decreased the plasma glucose level under starvation condition in diabetic rats.
